# Crystal structure of the co-crystal salt 2-amino-6-bromo­pyridinium 2,3,5,6-tetra­fluoro­benzoate

**DOI:** 10.1107/S2056989019001294

**Published:** 2019-01-31

**Authors:** Eric Bosch

**Affiliations:** a Department of Chemistry, Missouri State University, Springfield, MO 65897, USA

**Keywords:** crystal structure, 2-amino-6-bromo­pyridine, 2,3,5,6-tetra­fluoro­benzoic acid, 2-amino-6-pyridinium 2,3,5,6-tetra­fluoro­benzoate, hydrogen bond

## Abstract

The asymmetric unit of the co-crystal salt 2-amino-6-bromo­pyridinium 2,3,5,6-tetra­fluoro­benzoate, C_5_H_6_BrN_2_^+^·C_7_HF_4_O_2_^−^, contains one pyridinium cation and one benzoate anion. In the crystal, the amino­pyridinium cationic unit forms two hydrogen bonds to the benzoate oxygen atoms in an *R*_2_^2^(8) motif. Two pyridinium benzoate units are hydrogen bonded through self-complementary hydrogen bonds between the second amine hydrogen and a carboxyl­ate O with a second *R*_2_^2^(8) motif to form a discrete hydrogen-bonded complex containing two 2-amino-6-bromo­pyridinium moieties and two 2,3,5,6-tetra­fluoro­benzoate moieties. The 2-amino-6-bromo­pyridinium moieties π-stack in a head-to-tail mode with a centroid–centroid separation of 3.7227 (12) Å and adjacent tetra­fluoro­benzoates also π-stack in a head-to-tail mode with a centroid–centroid separation of 3.6537 (13) Å.

## Abstract 2

The title compound crystallized from an equimolar mixture of 2-amino-6-bromo­pyridine and 2,3,5,6-tetra­fluoro­benzoic acid in absolute ethanol.

## Chemical context   

**Figure 1:**
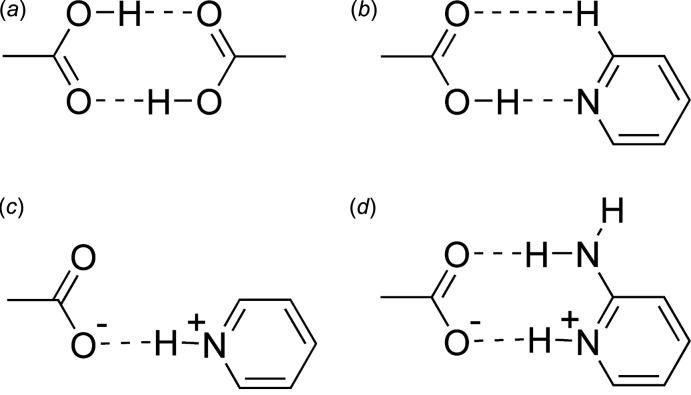
Potential hydrogen-bonding motifs for (*a*) carb­oxy­lic acid dimers, (*b*) neutral hydrogen-bonded pyridine carb­oxy­lic acid co-crystals, (*c*) charge-assisted pyridinium carboxyl­ate hydrogen-bonded complexes, and (*d*) charge-assisted 2-amino­pyridinium carboxyl­ate hydrogen-bonded complexes.

The fields of crystal engineering and supra­molecular chemistry rely on the identification and application of versatile synthons to guide the construction of mol­ecular solids (Desiraju, 1995 ▸, 2013 ▸). For example carb­oxy­lic acids are known to form a centrosymmetric dimer through self-complementary O—H⋯O hydrogen bonds (Fig. 1 ▸ *a*) in addition to hydrogen-bonded catemer chains and rings. It has been shown that these hydrogen bonds can be diverted by O—H⋯N hydrogen bonding to pyridines, often supported by a non-conventional pyridine C—H⋯O hydrogen bond (Fig. 1 ▸ *b*). The inter­action of the more basic pyridines, for example 4-(*N*,*N*-di­methyl­amino)­pyridine, with carb­oxy­lic acids most often yields charge-assisted hydrogen-bonded salts (Fig. 1 ▸ *c*). Similarly, the combination of 2-amino­pyridines and benzoic acids has been demonstrated to be a reliable supra­molecular synthon resulting in the formation of charge-assisted hydrogen-bonded complexes shown in Fig. 1 ▸ *d* (Bis & Zaworotko, 2005 ▸). The formation of hydrogen-bonded co-crystals or salts of amines and acids has potential in the pharmaceutical field where the physicochemical properties of active pharmaceuticals, including aqueous solubility and physical and chemical stabil­ity, may be modulated and tailored by co-crystal or salt formation (Schultheiss & Newman, 2009 ▸). For example a study involving the non-steroidal anti-inflammatory drug piroxicam reported the formation of 19 pyridine based co-crystals (Wales *et al.*, 2012 ▸). The present study presents the first co-crystal/salt formed between a substituted pyridine and 2,3,5,6-tetra­fluoro­benzoic acid.
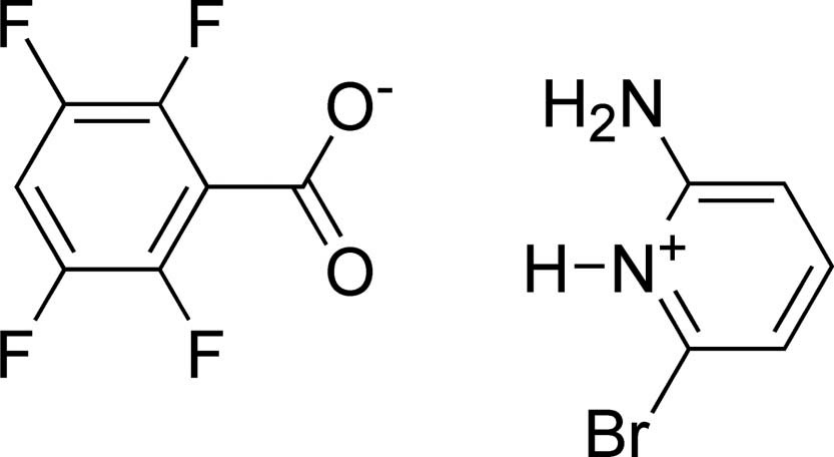


## Structural commentary   

**Figure 2:**
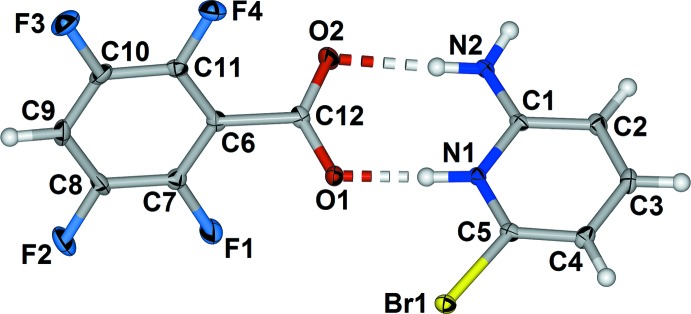
The mol­ecular structure of the co-crystal salt (I) showing the atom-labeling scheme. Displacement ellipsoids drawn at the 50% probability level and hydrogen bonds (Table 1 ▸) are shown as dotted lines.

The asymmetric unit of the co-crystal salt 2-amino-6-bromo­pyridinium 2,3,5,6-tetra­fluoro­benzoate (I), contains one pyridinium cation and one benzoate anion that are held together by two charge-assisted hydrogen bonds (Table 1 ▸, first two entries) to form an 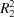
(8) motif (Fig. 2 ▸). The bond distance C12—O2 is slightly shorter than C12—O1, with distances of 1.236 (2) and 1.267 (2) Å respectively. The atoms that form this 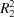
(8) motif (Fig. 1 ▸) are almost coplanar, with the maximum deviation above and below the least-squares plane calculated through all of these atoms being 0.169 (7) and −0.147 (8) Å, respectively, for O2 and O1. The angle between the planes defined by the benzene and pyridine rings is 67.04 (7)° and the carboxyl­ate anion is twisted out of the plane of the benzene ring, with C12 0.103 (3) Å above the plane of the benzene ring and O1 1.043 (3) Å above, and O2 0.713 (4) Å below the plane defined by the benzene ring.

## Supra­molecular features   

**Figure 3:**
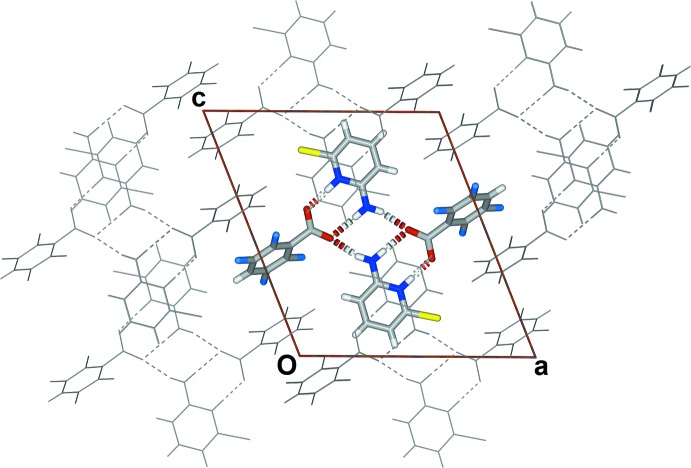
Part of the crystal structure of (I) viewed along *b*, highlighting the hydrogen-bonded dimeric salt unit.

In the co-crystal salt (I), adjacent amino pyridinium benzoate salt units are linked into dimeric salt complexes with self-complementary hydrogen bonds (Table 1 ▸, entry 3) from the second amine hydrogen atom and carboxyl­ate oxygen atom O2 in a second 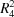
(8) motif (Fig. 3 ▸). The two components are relatively well separated within the crystal structure into zones parallel to the *c* axis.

**Figure 4:**
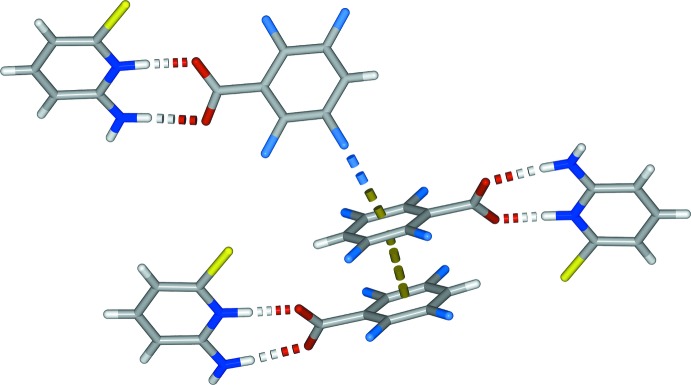
Partial view of the packing in the crystal structure of (I) highlighting the head-to-tail π-stacking of the tetra­fluoro­benzoate mol­ecules and the C—F⋯π inter­action.

There are two inter­actions that involve the tetra­fluoro­benzoate (Fig. 4 ▸). Adjacent tetra­fluoro­benzoates π-stack in a head-to-tail mode with a *Cg*1⋯*Cg*1^i^ distance of 3.6537 (13) Å [symmetry code: (i) −*x*, 1 − *y*, *z*; *Cg*1 is the centroid of the benzene ring C6–C11] and there is a close C—F⋯π inter­action with a *Cg*1⋯F3^ii^ distance of 3.1640 (17) Å [symmetry code: (ii) −*x*, *y* − 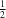, 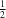 − *z*].

**Figure 5:**
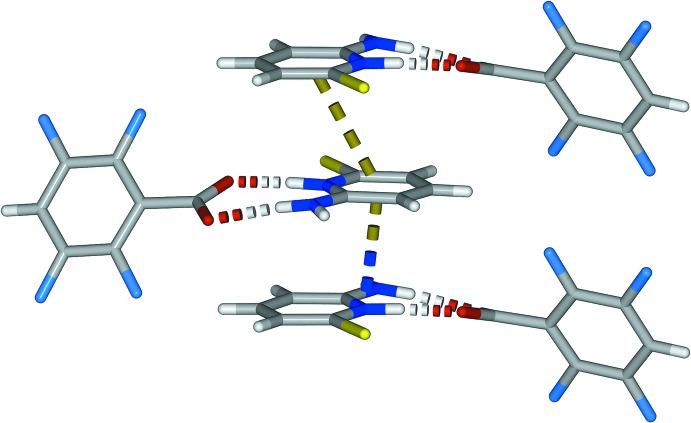
Partial view of the packing in the crystal structure of (I) highlighting the head-to-tail π-stacking of the 2-amino-6-bromo­pyridinium cations.

The 2-amino­pyridinium groups form offset alternating head-to-tail π-stacks parallel to the *b* axis (Fig. 5 ▸) with a *Cg*2⋯*Cg*2^iii^ distance of 3.7227 (12) Å and a shortest perpendicular inter­planar distance of 3.2547 (8) Å [symmetry code: (iii) 1 − *x*, *y* − 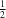, 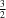 − *z*; *Cg*2 is the centroid of the pyridine ring].

There is one short contact to the bromine with a C9⋯Br1^iv^ distance of 3.867 (2) Å [symmetry code: (iv) 1 − *x*, 1 − *y*, 2 − *z*].

## Database survey   

A search of the Cambridge Crystallographic Database (Version 5.39, update of August 2018; Groom *et al.*, 2016 ▸) using *Conquest* (Bruno *et al.*, 2002 ▸) for structures including the neutral carb­oxy­lic acid dimer synthon as shown in Fig. 1 ▸ *a* revealed 6,016 hits, while a search for neutral pyridine carb­oxy­lic acid inter­actions where the distance between the acid proton and the pyridine N is equal to or less than the sum of the van der Waals radii revealed 2189 hits. In 966 of the 2189 structures the distance between the carbonyl O and the pyridine H is also equal to or less than the sum of the van der Waals radii, corresponding to the synthon shown in Fig. 1 ▸ *b*. A related search of the Cambridge Crystallographic Database for co-crystals with 4-(*N*,*N*-di­methyl­amino)­pyridine and carb­oxy­lic acids revealed only four neutral co-crystals and 54 structures corresponding to the pyridinium carboxyl­ate as shown in Fig. 1 ▸ *c*. A similar search for co-crystals formed between 2-amino­pyridines with benzoic acids yielded 41 hits, of which 40 feature charge-assisted amino­pyridinium carboxyl­ate hydrogen-bonded co-crystals as the result of proton transfer shown in Fig. 1 ▸ *d*. The structure that is reported to form a neutral hydrogen-bonded complex corresponds to the co-crystal formed between 2-amino­pyridine and 4-amino­benzoic acid [refcode WOPCOV; Chandrasekaran & Babu, 2014 ▸]. Finally there is only one reported co-crystal of 2,3,5,6-tetra­fluoro­benzoic acid, or the corresponding 2,3,5,6-tetra­fluoro­benzoate, with an organic base. In that example theophylline forms a neutral hydrogen-bonded complex (Corpinot *et al.*, 2016 ▸).

## Synthesis and crystallization   

2-Amino-6-bromo­pyridine and 2,3,5,6-tetra­fluoro­benzoic acid were used as supplied. An equimolar amount (0.1 mmol) of each component were added to a screw-capped vial and 3 mL of ethanol added to effect a clear colorless solution that was allowed to slowly concentrate over two weeks. A homogeneous mass of crystals was obtained.

## Refinement   

Crystal data, data collection and structure refinement details are summarized in Table 2 ▸. All hydrogen atoms were located in Fourier-difference maps. Hydrogen atoms involved in hydrogen-bonding inter­actions were restrained in the refinement with N—H = 0.87 (2) Å and with *U*_iso_(H) = 1.2*U*_eq_(N). The aromatic H atoms were included in the refinement at calculated positions with C—H = 0.95 Å and *U*_iso_(H) = 1.2*U*_eq_(C).

## Supplementary Material

Crystal structure: contains datablock(s) I, global. DOI: 10.1107/S2056989019001294/pk2612sup1.cif

Structure factors: contains datablock(s) I. DOI: 10.1107/S2056989019001294/pk2612Isup2.hkl

Supporting information file. DOI: 10.1107/S2056989019001294/pk2612Isup3.cml

CCDC reference: 1893117

Additional supporting information:  crystallographic information; 3D view; checkCIF report

## Figures and Tables

**Table 1:** Hydrogen-bond geometry (Å, °)

*D*—H⋯*A*	*D*—H	H⋯*A*	*D*⋯*A*	*D*—H⋯*A*
N1—H1⋯O1	0.91 (2)	1.68 (2)	2.585 (2)	177 (2)
N2—H2*A*⋯O2	0.87 (2)	1.98 (2)	2.845 (2)	175 (2)
N2—H2*B*⋯O2^i^	0.87 (2)	2.02 (2)	2.854 (2)	162 (2)
C9—H9⋯Br1^ii^	0.95	2.94	3.867 (2)	166

Symmetry codes: (i) 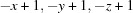; (ii) 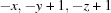.

**Table 2:** Experimental details

Crystal data	
Chemical formula	C_5_H_6_BrN_2_^+^·C_7_HF_4_O_2_^−^
*M*_r_	367.11
Crystal system, space group	Monoclinic, *P*2_1_/*c*
Temperature (K)	100
*a*, *b*, *c* (Å)	13.7230 (9), 6.5757 (4), 15.3224 (10)
β (°)	111.841 (1)
*V* (Å^3^)	1283.42 (14)
*Z*	4
Radiation type	Mo *K*α
μ (mm^−1^)	3.26
Crystal size (mm)	0.25 × 0.20 × 0.03

Data collection	
Diffractometer	Bruker APEXII CCD
Absorption correction	Multi-scan (*SADABS*; Bruker, 2014 ▸)
*T*_min_, *T*_max_	0.788, 1.000
No. of measured, independent and observed [*I* > 2σ(*I*)] reflections	16099, 2847, 2395
*R*_int_	0.045
(sin θ/λ)_max_ (Å^−1^)	0.641

Refinement	
*R*[*F* ^2^ > 2σ(*F* ^2^)], *wR*(*F* ^2^), *S*	0.026, 0.059, 1.04
No. of reflections	2847
No. of parameters	199
No. of restraints	3
H-atom treatment	H atoms treated by a mixture of independent and constrained refinement
Δρ_max_, Δρ_min_ (e Å^−3^)	0.41, −0.32

Computer programs: *SMART* and *SAINT* (Bruker, 2014 ▸), *SHELXT2018/2* (Sheldrick, 2015*a* ▸), *SHELXL2018/3* (Sheldrick, 2015*b* ▸) and *X-SEED* (Barbour, 2001 ▸).

## supplementary crystallographic information

### Crystal data

**Table:**

C_5_H_6_BrN_2_^+^·C_7_HF_4_O_2_^−^	*F*(000) = 720
*M**_r_* = 367.11	*D*_x_ = 1.900 Mg m^−^^3^
Monoclinic, *P*2_1_/*c*	Mo *K*α radiation, λ = 0.71073 Å
*a* = 13.7230 (9) Å	Cell parameters from 3850 reflections
*b* = 6.5757 (4) Å	θ = 2.7–25.7°
*c* = 15.3224 (10) Å	µ = 3.26 mm^−^^1^
β = 111.841 (1)°	*T* = 100 K
*V* = 1283.42 (14) Å^3^	Cut block, colourless
*Z* = 4	0.25 × 0.20 × 0.03 mm

### Data collection

**Table:**

Bruker APEXII CCD diffractometer	2847 independent reflections
Radiation source: fine-focus sealed tube	2395 reflections with *I* > 2σ(*I*)
Graphite monochromator	*R*_int_ = 0.045
Detector resolution: 8.3660 pixels mm^-1^	θ_max_ = 27.1°, θ_min_ = 1.6°
phi and ω scans	*h* = −17→17
Absorption correction: multi-scan (SADABS; Bruker, 2014)	*k* = −8→8
*T*_min_ = 0.788, *T*_max_ = 1.000	*l* = −19→19
16099 measured reflections	

### Refinement

**Table:**

Refinement on *F*^2^	3 restraints
Least-squares matrix: full	Hydrogen site location: mixed
*R*[*F*^2^ > 2σ(*F*^2^)] = 0.026	H atoms treated by a mixture of independent and constrained refinement
*wR*(*F*^2^) = 0.059	*w* = 1/[σ^2^(*F*_o_^2^) + (0.0271*P*)^2^ + 0.3891*P*] where *P* = (*F*_o_^2^ + 2*F*_c_^2^)/3
*S* = 1.04	(Δ/σ)_max_ = 0.002
2847 reflections	Δρ_max_ = 0.41 e Å^−^^3^
199 parameters	Δρ_min_ = −0.32 e Å^−^^3^

### Special details

**Table:**

Geometry. All esds (except the esd in the dihedral angle between two l.s. planes) are estimated using the full covariance matrix. The cell esds are taken into account individually in the estimation of esds in distances, angles and torsion angles; correlations between esds in cell parameters are only used when they are defined by crystal symmetry. An approximate (isotropic) treatment of cell esds is used for estimating esds involving l.s. planes.

### Fractional atomic coordinates and isotropic or equivalent isotropic displacement parameters (Å^2^)

**Table:**

	*x*	*y*	*z*	*U*_iso_*/*U*_eq_	
Br1	0.35106 (2)	0.41134 (3)	0.84202 (2)	0.01733 (7)	
F1	0.10223 (10)	0.15160 (19)	0.49369 (9)	0.0248 (3)	
F4	0.17441 (10)	0.8060 (2)	0.40322 (9)	0.0275 (3)	
F2	−0.10146 (10)	0.2028 (2)	0.38905 (10)	0.0321 (3)	
F3	−0.03037 (12)	0.8562 (2)	0.30158 (10)	0.0365 (4)	
O1	0.27555 (11)	0.4079 (2)	0.59997 (10)	0.0188 (3)	
O2	0.32636 (12)	0.4894 (3)	0.48172 (11)	0.0238 (4)	
N1	0.46382 (13)	0.4100 (3)	0.72562 (12)	0.0130 (4)	
H1	0.3977 (13)	0.404 (3)	0.6815 (14)	0.016*	
N2	0.53368 (14)	0.4068 (3)	0.61009 (13)	0.0171 (4)	
H2A	0.4701 (14)	0.424 (3)	0.5700 (14)	0.020*	
H2B	0.5859 (15)	0.422 (3)	0.5919 (16)	0.020*	
C4	0.57133 (17)	0.4158 (3)	0.88912 (15)	0.0170 (4)	
H4	0.577544	0.416389	0.952978	0.020*	
C2	0.65046 (17)	0.4148 (3)	0.77289 (15)	0.0172 (4)	
H2	0.711166	0.414715	0.757094	0.021*	
C12	0.25906 (16)	0.4564 (3)	0.51564 (15)	0.0155 (5)	
C5	0.47591 (16)	0.4124 (3)	0.81748 (14)	0.0147 (4)	
C1	0.54936 (16)	0.4114 (3)	0.70135 (15)	0.0136 (4)	
C7	0.07144 (17)	0.3268 (3)	0.44649 (15)	0.0173 (5)	
C3	0.66028 (17)	0.4183 (3)	0.86458 (15)	0.0182 (5)	
H3	0.728259	0.422448	0.912677	0.022*	
C11	0.10738 (17)	0.6527 (3)	0.40039 (15)	0.0183 (5)	
C10	0.00175 (18)	0.6802 (4)	0.34810 (15)	0.0228 (5)	
C8	−0.03368 (17)	0.3535 (4)	0.39179 (16)	0.0206 (5)	
C6	0.14445 (16)	0.4777 (3)	0.45227 (14)	0.0157 (5)	
C9	−0.06994 (18)	0.5308 (4)	0.34355 (15)	0.0241 (5)	
H9	−0.142601	0.549773	0.307970	0.029*	

### Atomic displacement parameters (Å^2^)

**Table:**

	*U*^11^	*U*^22^	*U*^33^	*U*^12^	*U*^13^	*U*^23^
Br1	0.01626 (12)	0.02221 (12)	0.01623 (12)	−0.00014 (9)	0.00917 (8)	0.00008 (9)
F1	0.0203 (7)	0.0188 (7)	0.0320 (8)	−0.0013 (5)	0.0061 (6)	0.0036 (6)
F4	0.0277 (8)	0.0257 (8)	0.0320 (8)	−0.0027 (6)	0.0144 (6)	0.0081 (6)
F2	0.0175 (7)	0.0384 (9)	0.0383 (9)	−0.0100 (6)	0.0081 (6)	−0.0041 (7)
F3	0.0341 (8)	0.0443 (9)	0.0320 (9)	0.0155 (7)	0.0133 (7)	0.0215 (7)
O1	0.0134 (7)	0.0282 (9)	0.0142 (8)	0.0001 (7)	0.0045 (6)	0.0028 (7)
O2	0.0150 (8)	0.0418 (10)	0.0171 (8)	−0.0004 (7)	0.0089 (7)	0.0003 (7)
N1	0.0101 (8)	0.0137 (9)	0.0154 (9)	0.0021 (7)	0.0051 (7)	0.0015 (7)
N2	0.0115 (9)	0.0234 (10)	0.0184 (10)	0.0008 (8)	0.0079 (8)	0.0027 (8)
C4	0.0196 (11)	0.0169 (11)	0.0134 (10)	0.0032 (9)	0.0049 (9)	−0.0012 (9)
C2	0.0135 (10)	0.0150 (11)	0.0231 (12)	0.0021 (9)	0.0070 (9)	0.0004 (9)
C12	0.0145 (11)	0.0167 (11)	0.0159 (11)	−0.0007 (8)	0.0062 (9)	−0.0057 (8)
C5	0.0153 (10)	0.0129 (10)	0.0173 (11)	0.0005 (9)	0.0077 (9)	0.0003 (9)
C1	0.0137 (10)	0.0093 (10)	0.0195 (11)	0.0009 (8)	0.0083 (8)	0.0018 (8)
C7	0.0174 (11)	0.0193 (11)	0.0154 (11)	0.0014 (9)	0.0062 (9)	−0.0003 (9)
C3	0.0143 (11)	0.0169 (11)	0.0191 (11)	0.0022 (9)	0.0011 (9)	−0.0012 (9)
C11	0.0192 (11)	0.0240 (12)	0.0152 (11)	−0.0010 (9)	0.0103 (9)	0.0006 (9)
C10	0.0226 (12)	0.0313 (14)	0.0159 (11)	0.0111 (11)	0.0088 (10)	0.0098 (10)
C8	0.0132 (11)	0.0312 (13)	0.0191 (12)	−0.0056 (10)	0.0080 (9)	−0.0057 (10)
C6	0.0150 (11)	0.0225 (12)	0.0118 (10)	0.0018 (9)	0.0075 (9)	−0.0036 (9)
C9	0.0129 (11)	0.0439 (16)	0.0144 (12)	0.0052 (10)	0.0037 (9)	0.0002 (10)

### Geometric parameters (Å, º)

**Table:**

Br1—C5	1.886 (2)	C4—C3	1.405 (3)
F1—C7	1.342 (2)	C4—H4	0.9500
F4—C11	1.355 (3)	C2—C3	1.361 (3)
F2—C8	1.349 (3)	C2—C1	1.413 (3)
F3—C10	1.345 (3)	C2—H2	0.9500
O1—C12	1.267 (2)	C12—C6	1.517 (3)
O2—C12	1.236 (2)	C7—C8	1.384 (3)
N1—C5	1.355 (3)	C7—C6	1.389 (3)
N1—C1	1.357 (3)	C3—H3	0.9500
N1—H1	0.909 (16)	C11—C10	1.383 (3)
N2—C1	1.334 (3)	C11—C6	1.383 (3)
N2—H2A	0.868 (16)	C10—C9	1.374 (3)
N2—H2B	0.866 (16)	C8—C9	1.371 (3)
C4—C5	1.360 (3)	C9—H9	0.9500
			
C5—N1—C1	120.04 (18)	F1—C7—C8	118.9 (2)
C5—N1—H1	118.4 (15)	F1—C7—C6	120.24 (19)
C1—N1—H1	121.5 (15)	C8—C7—C6	120.8 (2)
C1—N2—H2A	117.8 (16)	C2—C3—C4	120.9 (2)
C1—N2—H2B	120.3 (16)	C2—C3—H3	119.5
H2A—N2—H2B	119 (2)	C4—C3—H3	119.5
C5—C4—C3	117.10 (19)	F4—C11—C10	118.3 (2)
C5—C4—H4	121.4	F4—C11—C6	120.04 (19)
C3—C4—H4	121.4	C10—C11—C6	121.6 (2)
C3—C2—C1	119.5 (2)	F3—C10—C9	120.1 (2)
C3—C2—H2	120.2	F3—C10—C11	119.1 (2)
C1—C2—H2	120.2	C9—C10—C11	120.8 (2)
O2—C12—O1	126.5 (2)	F2—C8—C9	120.0 (2)
O2—C12—C6	118.28 (19)	F2—C8—C7	118.5 (2)
O1—C12—C6	115.18 (18)	C9—C8—C7	121.5 (2)
N1—C5—C4	123.21 (19)	C11—C6—C7	117.1 (2)
N1—C5—Br1	115.97 (15)	C11—C6—C12	121.12 (19)
C4—C5—Br1	120.82 (16)	C7—C6—C12	121.8 (2)
N2—C1—N1	117.94 (19)	C8—C9—C10	118.1 (2)
N2—C1—C2	122.87 (19)	C8—C9—H9	120.9
N1—C1—C2	119.18 (19)	C10—C9—H9	120.9

### Hydrogen-bond geometry (Å, º)

**Table:**

*D*—H···*A*	*D*—H	H···*A*	*D*···*A*	*D*—H···*A*
N1—H1···O1	0.91 (2)	1.68 (2)	2.585 (2)	177 (2)
N2—H2*A*···O2	0.87 (2)	1.98 (2)	2.845 (2)	175 (2)
N2—H2*B*···O2^i^	0.87 (2)	2.02 (2)	2.854 (2)	162 (2)
C4—H4···Br1^ii^	0.95	3.13	4.017 (2)	155
C9—H9···Br1^iii^	0.95	2.94	3.867 (2)	166

Symmetry codes: (i) −*x*+1, −*y*+1, −*z*+1; (ii) −*x*+1, −*y*+1, −*z*+2; (iii) −*x*, −*y*+1, −*z*+1.
